# Chemopreventive effects of celecoxib are limited to hormonally responsive mammary carcinomas in the *neu*-induced retroviral rat model

**DOI:** 10.1186/bcr1864

**Published:** 2008-02-15

**Authors:** Stephan Woditschka, Jill D Haag, Bob Mau, Ronald A Lubet, Michael N Gould

**Affiliations:** 1 McArdle Laboratory for Cancer Research, University of Wisconsin, 1400 University Avenue, Madison, WI 53706, USA; 2 Genomics Center of Wisconsin, University of Wisconsin, 4400 Genetics–Biotechnology Center Building, 425 Henry Mall Madison, WI 53706, USA; 3 National Cancer Institute, Division of Cancer Prevention, 6130 Executive Blvd, Bethesda, MD 20892, USA

## Abstract

**Introduction:**

While current breast cancer chemoprevention strategies using selective estrogen response modulators and aromatase inhibitors are quite successful, their effects are limited to hormonally responsive breast cancer. Hormonally nonresponsive breast cancer (including estrogen receptor-negative cancer) is associated with poor prognosis for patients, and few chemoprevention agents exist for this type of cancer. The cyclooxygenase-2 inhibitor celecoxib (Celebrex^®^) is a nonsteroidal anti-inflammatory drug and as such is a potential candidate for the prevention of hormonally nonresponsive breast cancer.

**Methods:**

The chemopreventive effects of celecoxib were evaluated in the *neu*-induced retroviral rat mammary carcinogenesis model, to assess the efficacy of celecoxib on hormonally responsive and hormonally nonresponsive mammary carcinomas.

**Results:**

Dietary celecoxib at 1,200 mg/kg diet was highly efficacious in the prevention of hormonally responsive mammary carcinomas in intact rats, decreasing tumor multiplicity by 56% (*P *< 0.0001) and by 74% (*P *= 0.0002) in two independent experiments. No significant effect was found, however, on hormonally nonresponsive mammary carcinomas of ovariectomized rats. Treatment with a combination diet, consisting of tamoxifen at 2 mg/kg diet and celecoxib at 1,200 mg/kg diet, reduced tumor multiplicity by 72% (*P *= 0.0002) in intact rats. This reduction was not statistically different from that observed with celecoxib alone. Furthermore, long-term treatment with celecoxib was not associated with reductions in tumor volume in either intact rats or ovariectomized rats. In contrast, tamoxifen treatment and the combination regimen caused significant reductions in tumor volumes in intact rats (*P *= 0.01 and *P *= 0.004, respectively). Consistent with these data, decreases in proliferation and increases in apoptosis were detected in tamoxifen-treated and combination diet-treated tumors. No such modulations were observed in celecoxib-treated tumors.

**Conclusion:**

The chemopreventive effects of celecoxib appear to be limited to modulations in multiplicity of hormonally responsive mammary carcinomas. The fact that no synergistic or additive effects were observed in combination diet-treated rats raises the question of whether celecoxib is suitable for the prevention of hormonally nonresponsive breast cancer or for use in combination therapy with selective estrogen response modulators or aromatase inhibitors.

## Introduction

Although significant advances have been made in the field of breast cancer prevention, the mortality and morbidity rates for this disease remain high. This may in part be due to the fact that most chemoprevention strategies to date (that is, selective estrogen response modulators (SERMs) and aromatase inhibitors) target estrogen, and are thus limited in their efficacy for hormonally responsive breast cancer. Malignancies that do not respond to hormone ablation therapy (hormonally nonresponsive breast cancers, including estrogen receptor (ER)-negative tumors) are associated with poor prognosis for the patient. Efforts therefore need to be concentrated on evaluating chemopreventive compounds specifically for this subset of tumors.

Cyclooxygenase-2 (COX-2) is an inducible enzyme involved in prostaglandin synthesis from arachidonic acid and plays a central role in inflammation. COX-2 overexpression is sufficient to induce tumorigenesis of mammary cancer [1] and its inhibition is thought to be the principle mechanism for cancer prevention by nonsteroidal anti-inflammatory drugs [2]. While COX-2 expression is virtually absent from normal mammary parenchyma, its overexpression is observed in roughly one-third of human breast cancers [3]. In her-2/neu-positive lesions, COX-2 overexpression is even more frequent and was observed in 43% of invasive breast carcinomas and in as many as 63% in ductal carcinomas *in situ *[4].

The COX-2 selective inhibitor celecoxib has been shown in preclinical studies to prevent hormonally responsive mammary tumors in carcinogen-induced rat models [5-7]. In addition, there is evidence that celecoxib prevents mammary tumors in transgenic mice [8,9] and in a human xenograft model [10], both of which are ER-negative models and are therefore considered hormonally nonresponsive.

The best documented mechanism for cancer prevention with celecoxib involves the downregulation of local estrogen biosynthesis by aromatase enzyme Cyp19. Both *in vitro *data [11,12] and *in vivo *data [13] show that COX-2 inhibition is associated with prostaglandin E_2 _reduction and suppression of aromatase activity. Furthermore, there is evidence that the nonselective nonsteroidal anti-inflammatory drug aspirin is efficacious for the prevention of hormone receptor-positive (ER-positive and progesterone receptor-positive) breast cancer but not hormone receptor-negative breast cancer [14]. These data combined suggest that one mechanism by which celecoxib prevents mammary cancer is through reductions in estrogen, which would limit its preventive potential to hormonally responsive tumors.

We investigated this estrogen reduction hypothesis using the *neu*-induced retroviral rat mammary carcinogenesis model [15]. This chemoprevention model offers two distinct hormonal configurations, intact and ovariectomized. The intact model produces both hormonally responsive and hormonally nonresponsive mammary carcinomas. The SERM tamoxifen generally causes a 50% reduction in tumor multiplicity in intact rats [15], which is similar to its efficacy in women [16]. In ovariectomized rats, all of the mammary carcinomas that develop are hormonally nonresponsive. Molecularly, this hormonal nonresponsiveness is characterized by reductions >75% in ER levels and reductions of nearly 90% in PR levels, as quantified using a cytosolic receptor-binding assay [17]. Prevention strategies using tamoxifen are not efficacious for these tumors.

The experiments presented in the current manuscript chiefly address the questions of whether celecoxib is suitable for the prevention of hormonally nonresponsive mammary carcinomas and whether the preventive effects of celecoxib extend beyond those of the SERM tamoxifen.

## Materials and methods

### Chemoprevention in *neu*-induced rats

All animal experiments were performed at our facility under protocols approved by the University of Wisconsin Medical School Animal Care and Use Committee. Virgin Wistar–Furth female rats were obtained from Harlan Sprague–Dawley (Madison, WI, USA) at 6 weeks of age. All rats were group housed in suspended wire cages and were maintained in a light/dark cycle of 12 hours, receiving Teklad lab meal (#8604, Teklad, Madison, WI, USA) and acidified water *ad libitum*.

After 1–2 weeks of acclimation, at approximately 50–60 days of age, all rats underwent retroviral infusion with the pJR*neu *vector, which induces mammary carcinogenesis by expressing the activated *her2/neu *oncogene. The construction and generation of the pJR*neu *retroviral vector have been previously described [18,19]. Details on retroviral gene transfer into the mammary epithelium of the laboratory rat [20] and the application of this technology for chemoprevention in the *neu*-induced retroviral rat carcinogenesis model [15] have also been published previously. In brief, a suspension of replication-defective amphotropic retrovirus containing the activated *neu *oncogene was infused into the central ducts of the 12 rat mammary glands. The rats for the intact model were infused with viral titers of 1 × 10^5 ^clone-forming units (CFU)/ml and 7.5 × 10^4 ^CFU/ml (Experiments 1a and 2a, respectively), and were left intact. The animals for the ovariectomized model received viral titers of 5 × 10^5 ^CFU/ml and 1 × 10^5 ^CFU/ml (Experiments 1b and 2b, respectively), and underwent a bilateral ovariectomy 2 days after the infusion.

At 4 days post infusion, the rats were randomly assigned to the treatment groups and the experimental diets were started. All animals were weighed and palpated for mammary tumors weekly. The studies were terminated 12 weeks post infusion for intact animals and 18 weeks post infusion for ovariectomized rats. At necropsy, the total number of carcinomas, the mammary gland locations, and the size of each mammary carcinoma were recorded. A minimal size criterion of 3 mm in the largest two dimensions of each mammary carcinoma was applied in order to be included in the analysis. Tumor volumes were calculated from three-dimensional measurements using the formula: volume = 0.5 × length × width × height.

### Chemopreventive agents

Tamoxifen was purchased from Sigma (St Louis, MI, USA) and celecoxib (LKT Laboratories, Inc., St Paul, MI, USA) was obtained through the NCI Division of Cancer Prevention Repository (Rockville, MD, USA). All experimental diets were dry mixed in Teklad 4% fat rodent meal (Teklad, Madison, WI, USA), which was also used as the control diet. Dietary tamoxifen was prepared as 2 mg/kg diet and celecoxib as 1,200 mg/kg diet. The combination diet contained full doses of both compounds. All diets were prepared fresh weekly, stored at -20°C and were fed daily.

### Proliferation and apoptosis

Upon necropsy, mammary carcinomas from Experiment 2 were collected, fixed and paraffin-embedded. Consecutive slices were stained with H & E for histological evaluation or were used for proliferation and apoptosis assays.

The proliferation index of mammary carcinomas was evaluated by KI-67 staining [21,22]. Immunohistochemical procedures were performed according to standard protocols using VP-K452 (Vector Labs, Burlingame, CA, USA) as the KI-67 primary antibody. Primary antibody binding was visualized by horseradish peroxidase-conjugated secondary antibody, the VECTASTAIN^® ^avidin–biotinylated enzyme complex Elite System (Vector Labs) and diaminobenzidine as the chromogen.

The apoptotic index of mammary carcinomas was evaluated by terminal deoxynucleotidyl transferase-mediated nick end-labeling [23] using the TdT-FragEL™ DNA Fragmentation Detection Kit QIA 33 (Calbiochem, Darmstadt, Germany). Slides were processed according to the manufacturer's recommendations. Approximately 1,000 cells per tumor in several random fields were evaluated by light microscopy for proliferating or apoptotic cells.

### Tissue generation for the quantitative real-time PCR assay

Fully differentiated mammary carcinomas from the control diet group of Experiment 1a, which had previously been flash frozen and stored at -80°C, were used for the mammary carcinoma tissue group.

For the mammary gland and ductal carcinoma *in situ *tissue groups, 6-week-old Wistar–Furth virgin female rats were obtained from Harlan Sprague–Dawley. The rats were initially housed and treated the same as the animals used in the prevention experiments. Rats designated for mammary gland tissues were housed in our animal facility until 9 weeks of age, when they were sacrificed. Lower abdominal and upper inguinal mammary glands were excised, flash frozen and stored at -80°C. Rats designated for *in situ *carcinoma tissue groups underwent retroviral infusion with the pJR*neu *vector at 7 weeks of age. A high viral titer of 1 × 10^7 ^CFU/ml was used to insure a high density of *in situ *carcinomas within the ductal tree structure of the lower abdominal mammary glands.

Two days post infusion a subset of the rats underwent a bilateral ovariectomy. At 15 days post infusion all rats were sacrificed. *In situ *carcinoma containing portions, distal to the lymph nodes within the lower abdominal mammary glands, were excised, flash frozen and stored at -80°C.

### Quantitative real-time PCR assay

Expression of COX-2 was measured by quantitative real-time PCR. Rat mammary glands, mammary carcinomas and mammary glands containing *in situ *carcinomas were homogenized in Tri-Reagent (Molecular Research Center, Inc., Cincinnati, OH, USA) using a Polytron tissue homogenizer. Whole RNA was extracted using a MagMAX™-96 for Microarrays Total RNA Isolation Kit (Ambion, Inc., Austin, TX, USA), following the manufacturer's directions. RNA was quantified using a ND-1000 Spectrophotometer (NanoDrop Technologies, Wilmington, DE, USA) and was reverse-transcribed using the SuperScript^® ^II Reverse Transcriptase Kit (Invitrogen, Carlsbad, CA, USA) following the manufacturer's instructions.

The rat COX-2 specific forward (GCTGATGACTGCCCAACTC) and reverse (CGGGATGAACTCTCTCCTCA) primers were designed using Primer3 software [24]. Expression levels of acidic ribosomal phosphoprotein PO 36B4 have been shown to be estradiol independent [25], and the rat 36B4 transcript was used as the endogenous control using forward (AGCTTTGGGCATCACCACTA) and reverse (CTCCCACCTTGTCTCCAGTC) primers. Quantitative real-time PCR was performed using a SYBR^® ^Green PCR Master Mix Kit (Applied Biosystems, Foster City, CA, USA) on a 7900HT Sequence Detector (Applied Biosystems) under default PCR conditions.

Data analyses were performed using SDS 2.2 software (Applied Biosystems). The cycle threshold (C_T_) values were determined at a manual threshold at which all real-time curves were in he exponential doubling phase. Technical replicates were averaged and the COX-2 expression values were normalized against endogenous control 36B4 expression values. Average COX-2 transcript concentrations were calculated for all tissues groups. The fold changes in COX-2 expression for mammary carcinoma and *in situ *carcinomas tissue groups were reported relative to mammary gland baseline expression.

### Statistical analysis

The number of carcinomas per animal at necropsy was modeled using generalized linear models assuming Poisson and binomial link functions. Differences due to diets were assessed by comparing the change in scaled deviance due to diet with the appropriate chi-squared distribution. If an overall significant effect of diet was found, each diet was compared with the control diet individually using a similar procedure.

The tumor volume, as a cubed dimension parameter, represents a skewed distribution in which very large tumors contribute disproportionately to the group averages. A Box–Cox transformation was performed [26], converting the raw tumor volumes into log_10_-transformed values. The log_10_-transformed data approximate a normal distribution. A two-sided *t *test was performed to obtain the *P *values for differences in tumor volume.

Statistical analysis for final body weight and for proliferation and apoptosis assays were performed by the Wilcoxon rank-sum (Mann–Whitney) test.

For the quantitative real-time PCR measurements, the mean C_T _values were calculated across technical duplicates. In each tissue group, the difference between COX-2 C_T _values and endogenous control 36B4 C_T _values were determined for 12 individual samples. A two-sided *t *test was used to determine the *P *values for each tissue group compared with the mammary gland group.

## Results

### Effects of tamoxifen, celecoxib and the combination regimen on tumor multiplicity

**Figure 1:**
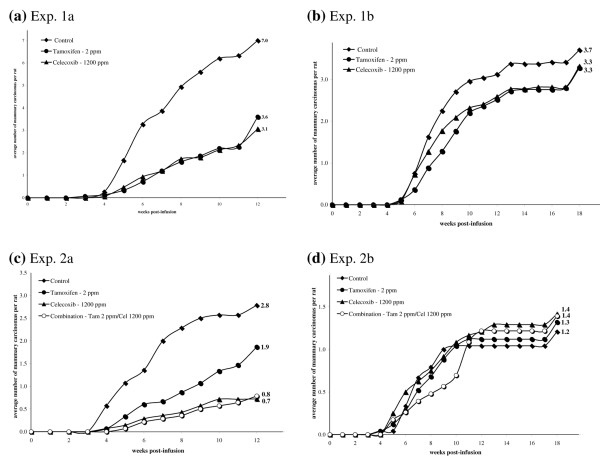
**Modulation of tumor multiplicity during treatment with tamoxifen and celecoxib alone and in combination**. Efficacies of **(a, b) **tamoxifen or celecoxib alone and **(c, d) **tamoxifen, celecoxib or combination treatment to prevent the development of mammary carcinomas in (a, c) intact rats and (b, d) ovariectomized rats. Tumor multiplicity curves labeled with the average number of mammary carcinomas per rat at the date of necropsy.

In the *neu*-induced rat mammary carcinogenesis model, approximately 50% of mammary carcinomas in the intact model are hormonally responsive and can therefore be prevented by tamoxifen (2 mg/kg diet) treatment. In two independent experiments using the intact model, the average number of mammary carcinomas developing per rat following tamoxifen treatment was decreased by 49% (*P *< 0.0001, Table 1 and Figure 1a) and by 33% (*P *= 0.1, Table 1 and Figure 1c), respectively. While the latter result did not reach statistical significance, there was a clear trend toward decreased tumor multiplicity throughout the course of the prevention experiment. In the ovariectomized model, where tumors are hormonally nonresponsive, tamoxifen treatment had no significant effect on tumor multiplicity compared with the control groups (Table 1 and Figure 1b,d).

**Table 1:** Chemopreventive effects of tamoxifen and celecoxib alone and in combination on tumor multiplicity, tumor volume and final body weights

Experiment	Intact/ovariectomized rat viral titer	Number of rats	Treatment	Tumor multiplicity (carcinomas/rat)	Tumor volume^a^, log_10 _(mean) (mm^3^)	Final body weight (g)
1a^b^	Intact, 1 × 10^5 ^CFU/ml	15	Control	7.0 ± 0.9	1.86 ± 0.06 (201)	208 ± 4.9
		15	Tamoxifen	3.6 ± 0.8, *P *< 0.0001	1.68 ± 0.07 (101), *P *= 0.03	184 ± 2.9, *P *< 0.0001
		15	Celecoxib	3.1 ± 0.8, *P *< 0.0001	1.96 ± 0.10 (444), ns	199 ± 2.9, ns
2a	Intact, 7.5 × 10^4 ^CFU/ml	14	Control	2.8 ± 0.7	2.16 ± 0.11 (433)	212 ± 2.3
		15	Tamoxifen	1.9 ± 0.8, ns	1.72 ± 0.13 (179), *P *= 0.01	186 ± 1.9, *P *< 0.0001
		13	Celecoxib	0.7 ± 0.2, *P *= 0.0002	1.98 ± 0.27 (438), ns	205 ± 2.7, ns
		14	Combination	0.8 ± 0.3, *P *= 0.0002	1.45 ± 0.18 (67), *P *= 0.004	181 ± 2.2, *P *< 0.0001
1b^b^	Ovariectomized, 5 × 10^5 ^CFU/ml	24	Control	3.7 ± 0.5	1.76 ± 0.08 (229)	261 ± 5.0
		25	Tamoxifen	3.3 ± 0.6, ns	1.77 ± 0.07 (207), ns	186 ± 2.0, *P *< 0.0001
		25	Celecoxib	3.3 ± 0.5, ns	1.84 ± 0.07 (511), ns	257 ± 2.3, ns
2b	Ovariectomized, 1 × 10^5 ^CFU/ml	25	Control	1.2 ± 0.2	1.60 ± 0.10 (88)	262 ± 3.1
		25	Tamoxifen	1.3 ± 0.2, ns	1.64 ± 0.12 (167), ns	188 ± 2.2, *P *< 0.0001
		23	Celecoxib	1.4 ± 0.3, ns	1.98 ± 0.11 (272), *P *= 0.03	250 ± 3.0, ns
		24	Combination	1.4 ± 0.5, ns	1.68 ± 0.10 (107), ns	182 ± 1.7, *P *< 0.0001

Mean values and standard errors of the means are indicated for tumor multiplicity, tumor volume and final body weights. CFU, clone-forming units. *P *values are indicated for the difference between the control and treatments for all measured parameters; ns, no significant difference. ^a^Calculated from three-dimensional measurements using the formula: volume = 0.5 × length × width × height. Data normalized by log_10 _transformation. ^b^Control and tamoxifen treatment groups have been published previously [15].

Celecoxib treatment was efficacious for the prevention of intact *neu*-induced rat mammary carcinomas, reducing tumor multiplicity in two independent trials by 56% (*P *< 0.0001, Table 1 and Figure 1a) and by 74% (*P *= 0.0003, Table 1 and Figure 1c). In ovariectomized rats, however, no significant reductions of tumor multiplicity were observed with celecoxib treatment (Table 1 and Figure 1b,d).

Regimens combining tamoxifen and celecoxib caused a significant reduction in tumor multiplicity in intact rats and generally paralleled the response caused by celecoxib treatment (*P *= 0.0003, Table 1 and Figure 1c). Similarly to tamoxifen and celecoxib individual treatments, the combination diet did not alter tumor multiplicity in ovariectomized rats.

### Effects of tamoxifen, celecoxib and the combination regimen on tumor volume

In two independent experiments, the administration of dietary tamoxifen caused significant decreases in the tumor volume in intact rats (*P *= 0.03 and *P *= 0.01, Table 1). No such effect was observed in ovariectomized rats.

Interestingly, celecoxib treatment did not reduce the tumor volume in either intact rats or ovariectomized rats. Indeed, a significant increase in tumor volume was seen in mammary carcinomas of ovariectomized rats following celecoxib treatment in Experiment 2b (*P *= 0.03, Table 1).

Similarly to tamoxifen-treated rats, treatment with the combination diet caused a significant decrease of the tumor volume in intact rats (*P *= 0.004, Table 1), while having no effect in ovariectomized animals.

### Effects of tamoxifen, celecoxib and the combination regimen on body weight gain

Tamoxifen treatment caused reductions in the final body weight of 12% in intact rats for Experiments 1a and 2a (*P *< 0.0001 and *P *< 0.0001, Table 1). This reduction was more pronounced in ovariectomized animals, where tamoxifen administration led to decreases in final body weight of 29% and of 28% in Experiments 1b and 2b (*P *< 0.0001 and *P *< 0.0001, Table 1), respectively.

In contrast, celecoxib treatment did not cause significant reduction in body weight gain in intact rats or ovariectomized rats (Table 1). Combination diet administration caused significant decreases in final body weights of 15% (*P *< 0.0001, Table 1) in intact rats and of 30% (*P *< 0.0001, Table 1) in ovariectomized animals, similar to those observed with tamoxifen treatment alone.

### Histopathological analysis

Histopathological evaluation of H & E-stained tumor sections classified all tumors as mammary carcinomas. No systematic morphological differences were associated with chemoprevention using tamoxifen, celecoxib or the combination regimen.

### Effects of tamoxifen, celecoxib and the combination regimen on proliferation and apoptosis in *neu*-induced mammary carcinomas

**Figure 2:**
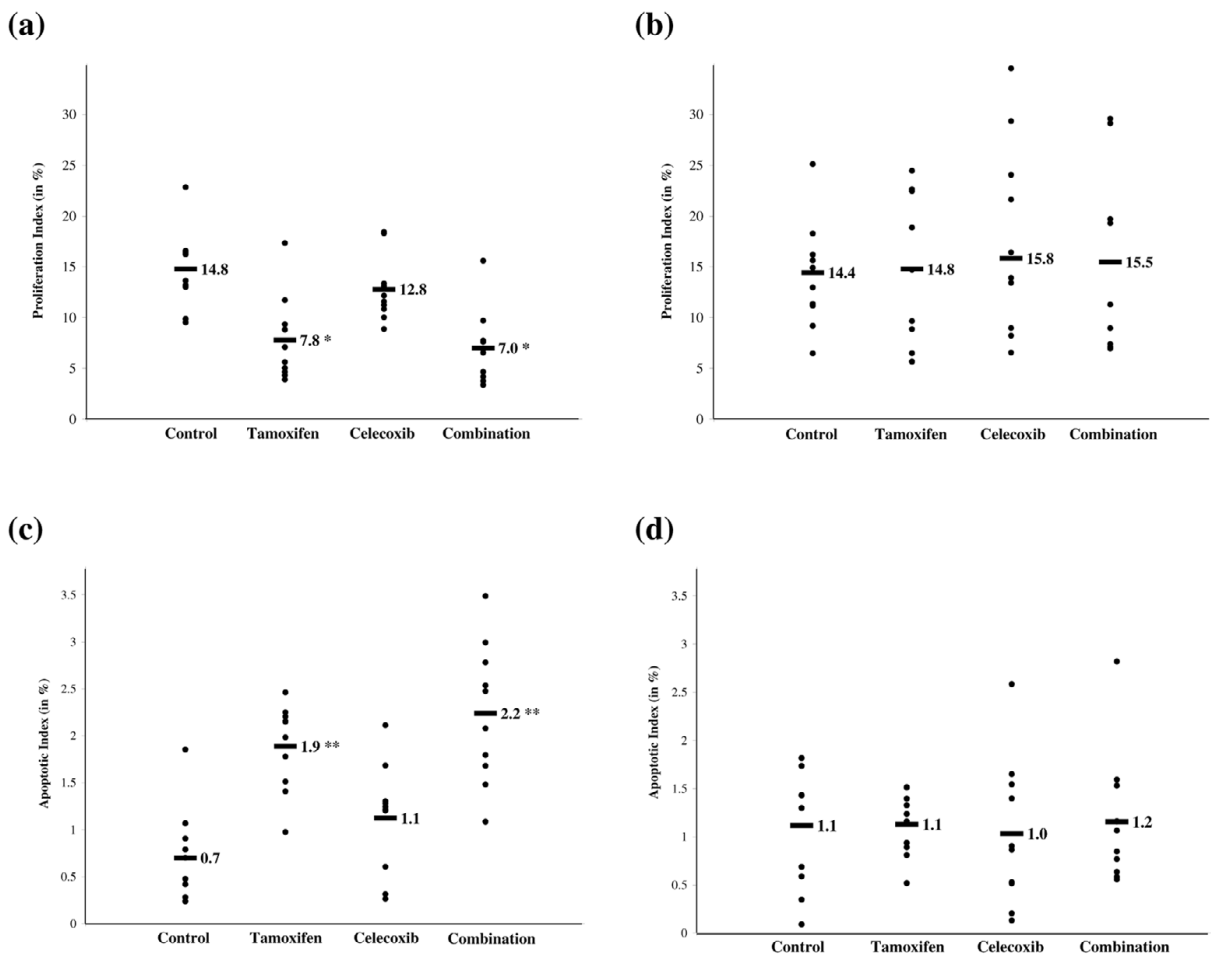
**Modulation of proliferation and apoptotic indices during tamoxifen and celecoxib treatment alone and in combination**. The proliferation index was measured by KI-67 expression in *neu*-induced mammary carcinomas from **(a) **intact rats and **(b) **ovariectomized rats. The terminal deoxynucleotidyl transferase-mediated nick end-labeling assay was performed to asses the apoptotic index in **(c) **intact rats and **(d) **ovariectomized rats. Ten mammary carcinomas were evaluated per treatment group. Approximately 1,000 cells were scored in each tumor. Labeled bars indicate means. *P < 0.005, **P < 0.001.

The long-term administration of tamoxifen resulted in a statistically significant reduction of the proliferation rate from 14.8% to 7.8% in intact rats (*P *= 0.004, Figure 2a). No such trend was observed for tumors from ovariectomized animals (Figure 2b). Tamoxifen treatment increased the apoptotic index of the *neu*-induced mammary carcinomas from 0.7% to 1.9% (*P *= 0.0008, Figure 2c) in intact rats. In the ovariectomized animals there was no measurable difference in the rate of apoptosis between control rats and tamoxifen-treated rats (Figure 2d).

Celecoxib treatment had no significant effect on the rates of proliferation or apoptosis of mammary carcinomas from either intact rats or ovariectomized rats (Figure 2).

The combination regimen treatment reduced the rate of proliferation of *neu*-induced mammary carcinomas in intact rats from 14.8% to 7.0% (*P *= 0.001, Figure 2a). The combination regimen also increased the apoptotic index from 0.7% to 2.2% (*P *= 0.0005, Figure 2c) in mammary carcinomas from intact rats. In mammary carcinomas from ovariectomized animals, the combination treatment did not modulate the rate of proliferation or apoptosis significantly (Figure 2b,d).

### Cyclooxygenase-2 mRNA expression in mammary gland, mammary carcinomas and *in situ *carcinomas

**Figure 3:**
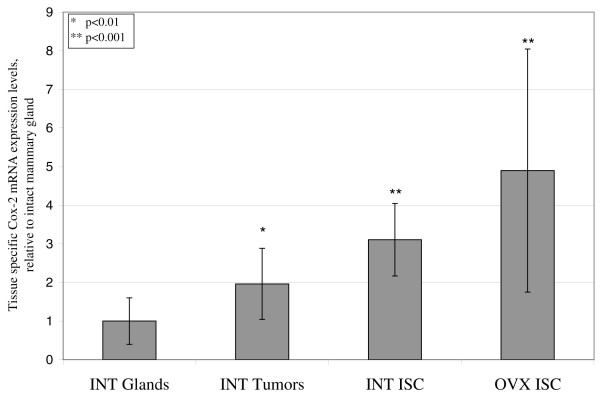
**Cyclooxygenase-2 mRNA expression in mammary gland, mammary carcinomas and *in situ *carcinomas**. Expression of cyclooxygenase-2 (COX-2) mRNA was measured by quantitative real-time PCR. Relative expression of COX-2 mRNA in mammary carcinomas and *in situ *carcinomas (ISC) in intact (INT) rats, as well as in ISC in ovariectomized (OVX) rats, was compared with baseline expression in normal mammary gland tissues. All expression values normalized to corresponding transcript expressions of rat acidic ribosomal phosphoprotein PO 36B4. The fold changes are shown. Error bars denote standard deviations. Significance levels are indicated.

COX-2 mRNA expression, as measured by quantitative real-time PCR assay, was lowest in normal mammary gland tissue, which was used as baseline expression and to which the COX-2 transcript levels of other tissues were compared. Fully differentiated mammary carcinomas in intact rats exhibited a mean 2.0-fold increased (*P *= 0.02) COX-2 mRNA expression (Figure 3). The COX-2 expression in *in situ *carcinomas of intact rats and ovariectomized rats was 3.1-fold and 4.9-fold increased (*P *= 0.0003 and *P *= 0.002, Figure 3), respectively. The difference between COX-2 expression in *in situ *carcinomas of intact rats and ovariectomized rats was not statistically different.

## Discussion

The chemopreventive effects of celecoxib appear to be limited to hormonally responsive mammary carcinomas in the *neu*-induced retroviral mammary carcinogenesis rat model. In two chemoprevention experiments with different control tumor multiplicities, celecoxib treatment proved ineffective in ovariectomized rats. These data suggest chemoprevention with celecoxib is dependent on an intact estrogen and progesterone milieu in this model.

Celecoxib's efficacy in intact *neu*-induced rats is inversely correlated to the control tumor multiplicity of the experiment, which, in our model, is a function of the retroviral titer. In a previous experiment in which the control animals had nearly 10 carcinomas per rat, celecoxib was not efficacious (data not shown). In the present Experiment 1a, however, it decreased tumor multiplicity by 50% at a control tumor multiplicity of seven carcinomas per rat. In Experiment 2, we further decreased the retroviral titer to evaluate whether this correlates with a greater chemopreventive effect of celecoxib. Indeed, at a very low control tumor multiplicity of less than three carcinomas per rat, celecoxib treatment resulted in a 74% reduction in tumor multiplicity.

The intact *neu*-induced retroviral rat model approximates the hormone responsiveness of breast cancer women. Similar to the 50% reductions in tumor multiplicity in tamoxifen-treated intact rats, the National Surgeon Adjuvant Breast and Bowel Project Breast Cancer Prevention Trial 1 concluded a 50% overall reduction of invasive breast cancer in women receiving tamoxifen [16]. The trial also estimated that about 70% of human breast cancers express ERα and therefore possess the potential to respond to hormone ablation therapy. In intact rats, in which both hormonally responsive and hormonally nonresponsive carcinomas arise, celecoxib treatment was associated with reductions of tumor multiplicity of 56% and 74%. It is therefore possible that tamoxifen and celecoxib both act on an overlapping subset of tumors, but celecoxib does so in a more comprehensive fashion, especially during conditions of optimized control tumor multiplicities. This hypothesis is also supported by the highly similar tumor multiplicities observed in intact rats treated with either celecoxib alone or with a combination diet.

The tumor volume, a measurement of the tumor growth rate, was modulated in intact rats by tamoxifen treatment and combination treatment, but not by celecoxib treatment. Mechanistically, these findings are supported by downregulation of proliferation indices and upregulation of apoptotic rates in treatments with tamoxifen and with the combination regimen. Celecoxib alone caused no such changes in *neu*-induced mammary carcinomas of intact rats or ovariectomized rats. The fact that celecoxib reduced tumor multiplicity but, unlike tamoxifen, failed to modulate tumor growth rates suggests a distinct mechanistic difference in the chemopreventive effects of those compounds. It should be noted that proliferation and apoptosis analysis was performed on long-term treated tumors and serves only to explain differences in the final tumor volume, and not as a measure of the prevention mechanism.

Consistent with the chemopreventive effects on hormonally responsive mammary carcinomas observed in our model, celecoxib has been shown highly efficacious for the prevention of mammary cancer in chemically induced rat models. Two different groups using 9,10-dimethylbenz-a-anthracene as the carcinogen in rats reported reductions in tumor multiplicity of 69% and of 86% associated with celecoxib administration at a dose of 1,500 ppm [5,6]. This effect is more dramatic than that observed in our model. In the 9,10-dimethylbenz-a-anthracene model, however, almost all tumors are hormonally responsive, resulting in prevention rates of >85% tumor multiplicity with tamoxifen treatment or ovariectomy [27]. The modulation of the tumor volume appears less consistent. While our finding that the tumor volume is not reduced with celecoxib treatment is substantiated in one study using the 9,10-dimethylbenz-a-anthracene rat model [6], another study reported an 81% reduction of the tumor volume using the same dose of 1,500 ppm [5].

Mammary tumors are ER-negative in mouse mammary tumor virus-*neu *transgenic mice, and therefore tend to be hormonally nonresponsive tumors. In contrast to our findings that celecoxib did not reduce tumor multiplicity of hormonally nonresponsive mammary carcinomas, tumor multiplicity was reduced by 41% in mouse mammary tumor virus-*neu *transgenic mice at a dose of 900 ppm [9]. This result should be understood in context, however, because tamoxifen treatment also has been shown to significantly decrease tumor multiplicity in this preclinical model [28]. Interestingly, no reductions in tumor volume were observed in celecoxib-treated mouse mammary tumor virus-*neu *transgenic mice [9]. This is consistent with our finding that celecoxib treatment does not modulate tumor volume in hormonally nonresponsive mammary carcinomas.

In a recent publication, the preventive effects of celecoxib on ER-positive and ER-negative breast cancers were investigated in a human xenograft mouse model [10]. Similar to our findings, proliferation as measured by Ki-67 expression was not modulated by celecoxib in either ER-positive or ER-negative xenografts. These experiments did, however, reveal a celecoxib-mediated increase in apoptosis, as measured by the terminal deoxynucleotidyl transferase-mediated nick end-labeling assay, in ER-positive xenografts only.

Importantly, no significant reductions in body weights were observed in celecoxib-treated animals. In contrast, tamoxifen treatment is associated with significant reductions in body weight gain in both intact and ovariectomized models at physiologically active doses. Although the utilized dose of tamoxifen caused decreases in the final body weight, the dose is nontoxic in the sense that doses of tamoxifen 50 times greater (100 ppm) did not cause lethality in intact rats (R.A. Lubet, unpublished data). Interestingly, even though tamoxifen treatment had a greater effect on the body weights of ovariectomized rats, this was not associated with a reduction in tumor multiplicity compared with control rats.

In general, COX-2 expression in the *neu*-induced retroviral mammary carcinogenesis rat models recapitulates the expression patterns observed in women. While rat mammary gland tissue expresses COX-2 at very low baseline levels, COX-2 overexpression is observed in mammary carcinomas and, to an even greater degree, in *in situ *carcinomas. This is consistent with the observation that COX-2 is not expressed in normal breast tissue but is overexpressed in 43% of her-2/*neu*-positive invasive breast carcinomas and in 63% of ductal carcinomas *in situ *[4].

Importantly, COX-2 is expressed in *in situ *carcinomas of ovariectomized rats in our model. The fact that celecoxib does not prevent mammary carcinomas in ovariectomized rats therefore appears to be independent of the COX-2 expression in these tissues.

The present observations are consistent with a mechanism by which celecoxib prevents mammary cancer through reductions in estrogen, limiting its preventive potential to hormonally responsive tumors.

## Conclusion

Overall, our data indicate that chemoprevention with celecoxib is limited to hormonally responsive mammary carcinomas and that this compound does not modulate the tumor volume in our model. The fact that no synergistic or additive effects were observed in combination diet-treated rats raises the question of whether celecoxib is suitable for the prevention of hormonally nonresponsive breast cancer or for use in combination therapy with SERMs or aromatase inhibitors. While selective cyclooxygenase-2 inhibitors might be generally thought a nonendocrine alternative to SERMs or aromatase inhibitors, we provided preclinical evidence that this might be an incomplete assessment that warrants further investigation.

## Abbreviations

CFU = clone-forming units; COX-2 = cyclooxygenase-2; ER = estrogen receptor; H & E = hematoxylin and eosin; PCR = polymerase chain reaction; ppm = parts per million; SERM = selective estrogen response modulator.

## Competing interests

The authors declare that they have no competing interests.

## Authors' contributions

SW carried out the prevention experiments, the immunoassays and the quantitative real-time PCR assay and drafted the manuscript. JDH assisted in designing and performing the prevention experiments and revised the manuscript. BM performed the statistical analysis for all parameters scored in the manuscript. RAL codesigned the prevention experiments and revised the manuscript. MNG conceived, codesigned, and coordinated the prevention experiments and revised the manuscript. All authors read and approved the final manuscript.
